# Kinetic Characterization and Catalytic Mechanism of *N*-Acetylornithine Aminotransferase Encoded by *slr1022* Gene from *Synechocystis* sp. PCC6803

**DOI:** 10.3390/ijms24065853

**Published:** 2023-03-19

**Authors:** Zhi-Min Li, Fumei Bai, Xiaoqin Wang, Congcong Xie, Yuting Wan, Yating Li, Jianping Liu, Zhimin Li

**Affiliations:** 1 College of Chemistry and Materials, Jiangxi Agricultural University, Nanchang 330045, China; 2 Jiangxi Engineering Laboratory for the Development and Utilization of Agricultural Microbial Resources, College of Bioscience and Bioengineering, Jiangxi Agricultural University, Nanchang 330045, China; 3 Collaborative Innovation Center of Postharvest Key Technology and Quality Safety of Fruits and Vegetables in Jiangxi Province, Jiangxi Agricultural University, Nanchang 330045, China

**Keywords:** *N*-acetylornithine aminotransferase, site-directed mutagenesis, kinetic characterization, catalytic mechanism, *Synechocystis* sp. PCC6803

## Abstract

The enzyme encoded by *slr1022* gene from *Synechocystis* sp. PCC6803 was reported to function as *N*-acetylornithine aminotransferase, γ-aminobutyric acid aminotransferase, and ornithine aminotransferase, which played important roles in multiple metabolic pathways. Among these functions, *N*-acetylornithine aminotransferase catalyzes the reversible conversion of *N*-acetylornithine to *N*-acetylglutamate-5-semialdehyde with PLP as cofactor, which is a key step in the arginine biosynthesis pathway. However, the investigation of the detailed kinetic characteristics and catalytic mechanism of Slr1022 has not been carried out yet. In this study, the exploration of kinetics of recombinant Slr1022 illustrated that Slr1022 mainly functioned as *N*-acetylornithine aminotransferase with low substrate specificity to γ-aminobutyric acid and ornithine. Kinetic assay of Slr1022 variants and the model structure of Slr1022 with *N*-acetylornithine-PLP complex revealed that Lys280 and Asp251 residues were the key amino acids of Slr1022. The respective mutation of the above two residues to Ala resulted in the activity depletion of Slr1022. Meanwhile, Glu223 residue was involved in substrate binding and it served as a switch between the two half reactions. Other residues such as Thr308, Gln254, Tyr39, Arg163, and Arg402 implicated a substrate recognition and catalytic process of the reaction. The results of this study further enriched the understanding of the catalytic kinetics and mechanism of *N*-acetylornithine aminotransferase, especially from cyanobacteria.

## 1. Introduction

Pyridoxal 5′-phosphate (PLP), one of the active forms of vitamin B6, was first identified as a cofactor for transamination in the 1940s [1]. Due to versatile reactions catalyzed by PLP-dependent enzymes, it was hard to categorize these enzymes into evolutionary families based on amino acid sequences’ homology and reacting positions [2]. Many efforts had been made to explore the diverse reaction specificity and catalytic mechanisms of PLP-dependent enzymes in the past decades. Recently, PLP-dependent enzymes were classified into seven fold types based on three-dimensional structures [3] and their structure–function relationships were well reviewed and summarized [4].

Aminotransferases (Ats, EC 2.6.1.-), a big family divided into four classes from type I and type IV PLP-dependent enzymes, catalyze the reversible transamination between amino acids and α-keto acids [5]. The Ats utilizing substrates such as *N*-acetylornithine (AcOrn), ornithine (Orn), γ-aminobutyric acid (GABA), 7,8-diaminopelargonic acid, and ω-amino acid constitute type I class II PLP-dependent enzymes [5]. The functional characterization, catalytic mechanisms, and structural features of class II ATs from various organisms were well studied. For example, the human GABA aminotransferase (GABA-AT), one enzyme of the GABA shunt, transaminates GABA to succinic semialdehyde and controls the level of GABA [6]. Ornithine aminotransferase (OAT) catalyzes the reversible transamination of Orn to glutamate-5-semialdehyde with a coinstantaneous conversion of α-ketoglutarate (α-KG) to glutamate and it serves as an important enzyme involved in multiple metabolic pathways [7]. *N*-acetylornithine aminotransferase (AcOAT) transforms AcOrn into *N*-acetylglutamate-5-semialdehyde reversibly, which is a key step in the arginine biosynthesis pathway [8]. All ATs share a similar “ping-pong” catalytic mechanism, requiring two coupled half reactions to complete a transamination cycle [9]. In the first half reaction, a Lys residue in the active center attacks the aldehyde group of PLP to form an internal aldimine, which then reacts with amino group of one substrate to produce a ketimine. Thereafter, the keto acid product and pyridoxamine 5′-phosphate (PMP) are released by the hydrolysis of ketimine [5]. In the second half reaction, the reverse of the first half essentially, one keto acid substrate reacts with PMP to produce amino acid and regenerate PLP [5]. The amino acid sequences’ homology of class II ATs is quite low, whereas they preserve similar structural features and hold conserved residues in the active center [5]. For instance, the aforementioned Lys residue, which forms the Schiff base with PLP, is absolutely conserved among all ATs. Several other residues are highly conserved in class II ATs [5].

There are abundant studies about class II ATs from varieties of organisms, the studies on AcOAT are relatively few though. AcOAT from *Pseudomonas aeruginosa* was first reported to play a dual role in arginine biosynthesis and catabolism [10]. The isolation and characterization of AcOAT from *E. coli* were then reported [11]. The substrate specificity and inhibitor binding of AcOAT from *Salmonella typhimurium* were studied through crystal structure analysis [12]. The ORF of *slr1022* gene from *Synechocystis* sp. PCC6803 was identified to be a putative gene encoding AcOAT in 2000 [13], and this enzyme might also function as an OAT based on bioinformatic analysis [14]. More recently, this Slr1022 protein proved to function as GABA-AT and contributed to closing the tricarboxylic acid cycle of *Synechocystis* sp. PCC6803 via the GABA shunt [15], and the catalytic functions of recombinant Slr1022 as AcOAT and GABA-AT were confirmed by an enzymatic assay [16]. However, the detailed kinetic properties of Slr1022 were not characterized and the residues involved in the substrate recognition and catalysis of Slr1022 as AcOAT have not been explored yet. Here, the catalytic constants of recombinant Slr1022 as AcOAT, OAT, and GABA-AT were determined and the kinetic properties of Slr1022 variants and model structure of Slr1022 with the AcOrn-PLP complex were investigated to reveal the residues involved in substrate binding and catalysis. To the best of our knowledge, this study was the first to report the detailed kinetic characteristics of AcOAT from cyanobacteria.

## 2. Results

### 2.1. Oligomer State of Recombinant Slr1022 Protein

SDS-PAGE showed that the recombinant Slr1022 protein was eluted with the imidazole concentration of 100 mmol/L and the purity of the collected protein could be over 95% (Figure S1A). Gel filtration was used to determine the molecular weight of the recombinant Slr1022 protein in native state. As shown in Figure S1B,C, the molecular weight of the native protein (Mw) and its retention time (RT) in the high-resolution gel column were fitted well to obtain the equation: lnMw = 11.34 − 0.29 × RT. Since the retention time of Slr1022 protein was 23.84 min after passing through the FPLC system, the molecular weight of the native Slr1022 protein was calculated to be 83.9 kDa, which was 1.7 times the molecular weight of its monomer (49 kDa) [17]. Therefore, it was reasonable to conclude that the native Slr1022 protein existed in the form of dimer, which was consistent with the results of AcOATs from *Salmonella typhimurium* and *Mycobacterium tuberculosis* [12,18].

### 2.2. Kinetic Characterization of Recombinant Slr1022 Protein towards Various Substrates

**Figure 1:**
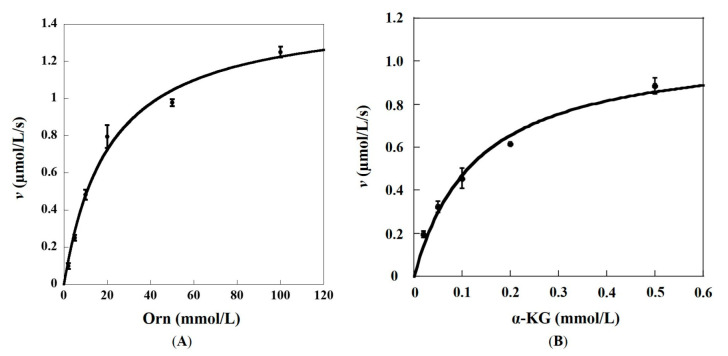
Kinetic profiles of recombinant Slr1022 protein as OAT. (**A**): Plot of the initial velocities as function of Orn concentrations, the concentration of α-KG was fixed at 0.5 mmol/L. (**B**): Plot of the initial velocities as function of α-KG concentrations, the concentration of Orn was fixed at 100 mmol/L. Data are expressed as the mean ± standard deviation, n = 3.

AcOAT is one member of the aminotransferase family, in which OAT and GABA-AT are also categorized [19]. These three aminotransferases catalyze the reversible transfer of the amino group of their respective substrates to the other substrate of α-keto acid, such as α-KG, with nearly the same catalytic mechanism [20]. Therefore, the kinetic properties of recombinant Slr1022 towards AcOrn, Orn, and GABA were characterized. The kinetic constants of recombinant Slr1022 as AcOAT were reported previously, as shown in Table 1 [17]. The kinetic parameters of recombinant Slr1022 as OAT were determined with Orn and α-KG as substrates. When the concentration of α-KG was fixed at 0.5 mmol/L, the *V*_max_ and *K_M_* of Orn with Slr1022 were fitted to be 1.48 ± 0.09 μmol/(L·s) and 20.9 ± 3.5 mmol/L, respectively (Figure 1A and Table 1). Therefore, the *k*_cat_ and *k*_cat_/*K_M_* of Orn with Slr1022 were calculated to be 0.16 ± 0.01 s^−1^ and 7.7 mol/(L·s), respectively (Table 1). When the concentration of Orn was fixed at 100 mmol/L, the *V*_max_ and *K_M_* of α-KG with Slr1022 were fitted to be 1.08 ± 0.09 μmol/(L·s) and 0.13 ± 0.03 mmol/L, respectively (Figure 1B and Table 1). Therefore, the *k*_cat_ and *k*_cat_/*K_M_* of α-KG with Slr1022 were calculated to be 0.11 ± 0.01 s^−1^ and 8.46 × 10^2^ mol/(L·s), respectively (Table 1).

**Figure 2:**
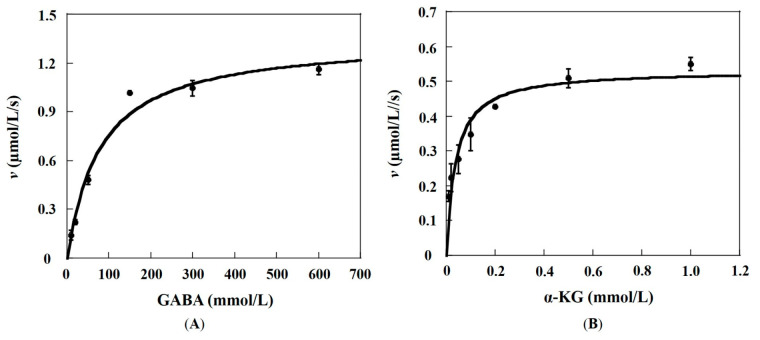
Kinetic profiles of recombinant Slr1022 protein as GABA-AT. (**A**): Plot of the initial velocities as function of GABA concentrations, the concentration of α-KG was fixed at 0.5 mmol/L. (**B**): Plot of the initial velocities as function of α-KG concentrations, the concentration of GABA was fixed at 600 mmol/L. Data are expressed as the mean ± standard deviation, n = 3.

The kinetic parameters of recombinant Slr1022 as GABA-AT were determined with GABA and α-KG as substrates. When the concentration of substrate α-KG was fixed at 0.5 mmol/L, the *V*_max_ and *K_M_* of GABA with Slr1022 were fitted to be 1.35 ± 0.10 μmol/(L·s) and 78.1 ± 18.7 mmol/L, respectively (Figure 2A and Table 1). Therefore, the *k*_cat_ and *k*_cat_/*K_M_* of GABA with Slr1022 were calculated to be 0.14 ± 0.01 s^−1^ and 1.8 mol/(L·s), respectively (Table 1). When the concentration of GABA was fixed at 600 mmol/L, the *V*_max_ and *K_M_* of α-KG with Slr1022 were fitted to be 0.53 ± 0.03 μmol/(L·s) and 0.04 ± 0.01 mmol/L, respectively (Figure 2B and Table 1). Therefore, the *k*_cat_ and *k*_cat_/*K_M_* of α-KG with Slr1022 were calculated to be 0.056 ± 0.03 s^−1^ and 1.4 × 10^3^ mol/(L·s), respectively (Table 1). 

### 2.3. Effects of Metal Ions and Temperature on Catalytic Activity of Slr1022 as AcOAT

**Figure 3:**
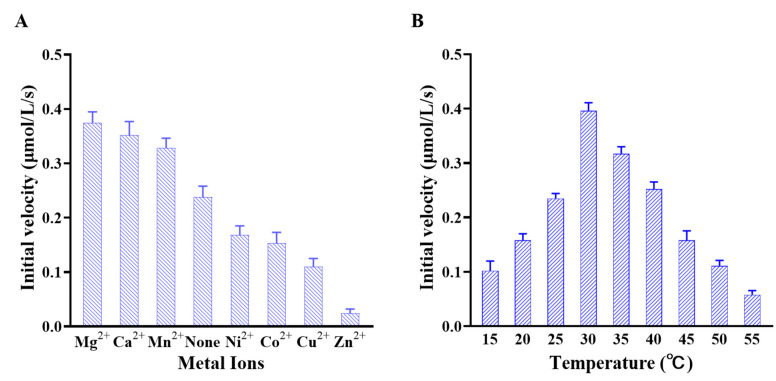
Effects of metal ions and temperature on AcOrn transaminase activity of Slr1022. (**A**): Effects of metal ions on the activity of Slr1022; (**B**): Temperature effect on the activity of Slr1022. Data are expressed as the mean ± standard deviation, n = 3.

The effect of metal ions (Mg^2+^, Ca^2+^, Mn^2+^, Ni^2+^, Co^2+^, Cu^2+^, and Zn^2+^) on the catalytic activity of Slr1022 as AcOAT was determined by measuring the initial reaction velocities. As shown in Figure 3A, compared to the initial velocity of the reaction solution with an addition of EDTA, which would chelate with metal ions to deplete the metal ions in the reaction solution, the addition of Mg^2+^, Ca^2+^, or Mn^2+^ would enhance the initial reaction velocities by 38–57%, whereas the addition of Ni^2+^, Co^2+^, or Cu^2+^ would decrease the initial velocities of enzymatic reaction by 29–54%. It is worth noting that the addition of Zn^2+^ would decrease the initial velocity to 10% of that of without metal ion. 

The temperature effect on the catalytic activity of Slr1022 as AcOAT was detected in the range of 15 °C to 55 °C. As shown in Figure 3B, the initial reaction velocities increased with the increasing temperature between 15 °C and 30 °C. Nevertheless, the initial velocities decreased with the increasing temperature between 30 °C and 55 °C. Therefore, the optimal temperature for Slr1022 as AcOAT was 30 °C (Figure 3B). As a result, the enzymatic activities of Slr1022 as AcOAT at 15 °C and 55 °C were only, respectively, 26% and 15% of that at the optimal temperature (Figure 3B). 

### 2.4. Structure Modeling and Model Quality Evaluation of Slr1022

**Figure 4:**
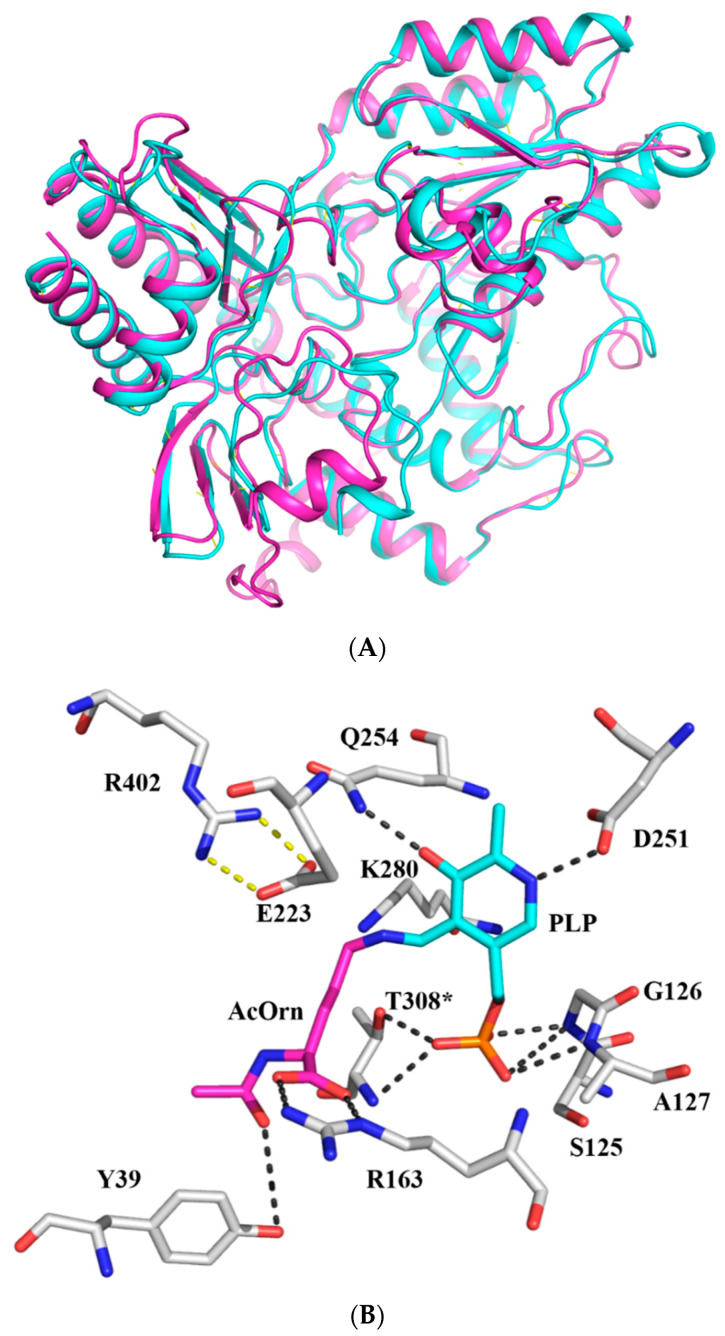
The overlapped cartoon and active site view of Slr1022 model structure. (**A**): Overlapped cartoon structure of Slr1022. The model structures from RoseTTAFold and SWISS-MODEL were colored as magenta and cyan, respectively. (**B**): Active site view of Slr1022 model structure generated from RoseTTAFold. The carbon of PLP, AcOrn, and amino acid residues were colored as cyan, magenta, and grey, respectively. Nitrogen, oxygen, and phosphor atoms were colored as blue, red, and orange, respectively. The hydrogen bonds were displayed as black dashed lines with distances less than 3.5 angstroms and the salt bridge interactions were shown as yellow dashed lines. The asterisk residue belonged to the neighboring subunit.

Amino acid sequences’ alignment showed that the amino acid sequences homology between Slr1022 and AcOATs from other sources, such as *Thermotoga maritima* (PDB ID: 2E54), *Stenotrophomonas maltophilia* (PDB ID: 6W7X), *Aquifex aeolicus* VF5 (PDB ID: 2EH6), and *Salmonella typhimurium* (PDB ID: 2PB0) [12], whose crystal structures were available, was lower than 50%, whereas the amino acid residues in the active center were highly conserved [17]. As a result, the model structure of Slr1022 was calculated by the online RoseTTAFold server and modeled by the online SWISS-MODEL server with a crystal structure of AcOAT from *Thermotoga maritima* (PDB ID: 2E54) as a template. There was no considerable deviation between the model structures from the RoseTTAFold and SWISS-MODEL server (Figure 4A). Furthermore, the RMS deviations between the two model structures were only 0.828, which indicated that these two model structures were intrinsically identical. After overlapping the model structure of Slr1022 with the crystal complex structure of PLP and AcOAT from *Thermus thermophilus* HB8 (PDB ID: 1WKG), the cofactor PLP was found to be located in the active pocket and surrounded by the residues in the active center (Figure 4B). In the crystal complex structure of AcOAT from *Thermus thermophilus* HB8, the cofactor PLP was covalently bound to AcOrn to form aldimine/ketimine intermediate. In the model complex structure of Slr1022 with the AcOrn-PLP intermediate, the nitrogen atom and hydroxyl group of the pyridine ring of PLP were hydrogen bonded to Asp251 and Gln254 with the distances of 2.6 Å and 2.6 Å, respectively (Figure 4B). The oxygen atoms of the phosphate group of PLP were within the range of hydrogen bond distances with the amide nitrogen of Ser125, Gly126, and Ala127 of the backbone (Figure 4B). Another hydrogen bond was provided by the oxygen atom of the phosphate group of PLP with the hydroxyl group of the Thr308 residue from the second subunit. Glu223, Arg163, Arg402, and Tyr39 residues were located in the vicinity of the intermediate complex, which might play a role in substrate recognition and catalysis (Figure 4B).

The Ramachandran plot of the Slr1022 model structure was generated by the PROCHECK program. The dark to light color in each region represented the most favored regions, the additional allowed regions, the generously allowed regions, and the disallowed regions (Figure S2). As shown in Figure S2 and Table S2, 86.2% of amino acid residues of the Slr1022 model structure were in the most favored regions and only 0.6% of residues were in the disallowed area. Together, 99.4% of the residues were in the allowed area. The model structure of Slr1022 was assessed by the Verify 3D program (Figure S3). The results showed that 89.92% of the amino acid residues were greater than or equal to 0.2 by 3D-1D scoring function, which indicated that the Slr1022 model structure passed the Verify 3D evaluation and was considered reasonable.

### 2.5. AcOrn Transaminase Activity Assay of Slr1022 Variants

The enzymatic kinetics of Slr1022 mutants were determined to infer the possible functions of amino acid residues in the active center. The kinetic parameters of the Slr1022 wild-type and mutants were summarized in Table 2. The *K_M_* of AcOrn and α-KG with T308A mutant were 0.14 mmol/L and 0.025 mmol/L, respectively, which were close to that of these two substrates with the Slr1022 wild-type. However, the catalytic rate (*k*_cat_) of T308A was only 0.3% of that of the wild-type Slr1022 (Table 2). These results indicated that the Thr308 residue functioned in the catalysis of reaction, not in the binding of substrates to the enzyme. The T308A mutation destroyed the hydrogen bond between the oxygen of the phosphate group of the cofactor PLP and the hydroxyl group of the side chain of Thr308. As a result, this mutation weakened the stability of PLP and affected the catalytic rate of reaction. Similar kinetic constants were obtained for the Q254A mutant, except that the catalytic rate was 0.9% of that of wild-type Slr1022 (Table 2). Therefore, the Gln254 residue played similar functions as that of the Thr308 residue. The Q254A mutation undermined the hydrogen bond between the hydroxyl group of PLP and the amino group of the side chain of the Gln254 residue. 

It was reported that the residues of Ser125, Gly126, and Ala127 (the numbers of residues were from *Synechocystis* sp. PCC6803) were conserved in the PLP-dependent aminotransferase family [17] and the above three residues from *Salmonella typhimurium* formed hydrogen bonds with oxygen of the phosphate group of PLP to enhance the stability of PLP [12]. As shown in Table 2, the G126A mutant demonstrated no catalytic activity at all, meanwhile the catalytic rates of S125A and A127S mutants were only 14% and 9.1% of those of the wild-type Slr1022. These results indicated that the residues of Ser125, Gly126, and Ala127 were important for catalysis. The mutation of Gly126 to Ala might have resulted in a steric hindrance of the active site, which prevented PLP from entering the active site and catalyzing the reaction.

There was no detectable catalytic activity of D251A and K280A mutants (Table 2). However, the D251E mutant retained 20% of the activity of that of the wild-type Slr1022. These results demonstrated that the carboxyl group of Asp251 and amino group of Lys280 were important for Slr1022 to function as AcOAT.

The mutation of Glu223 to Ala or Ser would increase the *K_M_* of AcOrn with the enzyme by 65 or 73 times that of the wild-type protein, respectively. However, the *K_M_* of α-KG with the enzyme and the catalytic rates of E223A and E223S were close to those of the wild-type protein (Table 2). These results proved that Glu223 residue was crucial for the binding of substrate AcOrn with enzyme and less important for the binding of substrate α-KG with enzyme, which was observed for the OAT from humans [21]. Similarly, the *K_M_* of AcOrn with enzyme would increase significantly by 4100 and 2080 folds for R163A and R402A mutants, respectively, compared to that of the wild-type protein. At the same time, the catalytic rates of R163A and R402A were 9.5% and 18% of those of wild-type Slr1022, respectively (Table 2). These results indicated that Arg163 and Arg402 residues not only functioned in substrate AcOrn binding, but also played a role in the catalytic turnover step. Likewise, the mutation of Tyr39 to Phe increased the *K_M_* of AcOrn with the enzyme to 0.47 ± 0.05 mmol/L, which was four times higher than that of the wild-type Slr1022. The catalytic rate of Y39F was only 7% of that of the wild-type protein (Table 2). These results demonstrated that Tyr39 residue participated in substrate binding and catalysis.

### 2.6. Spectroscopic Characterization of Slr1022 as AcOAT

**Figure 5:**
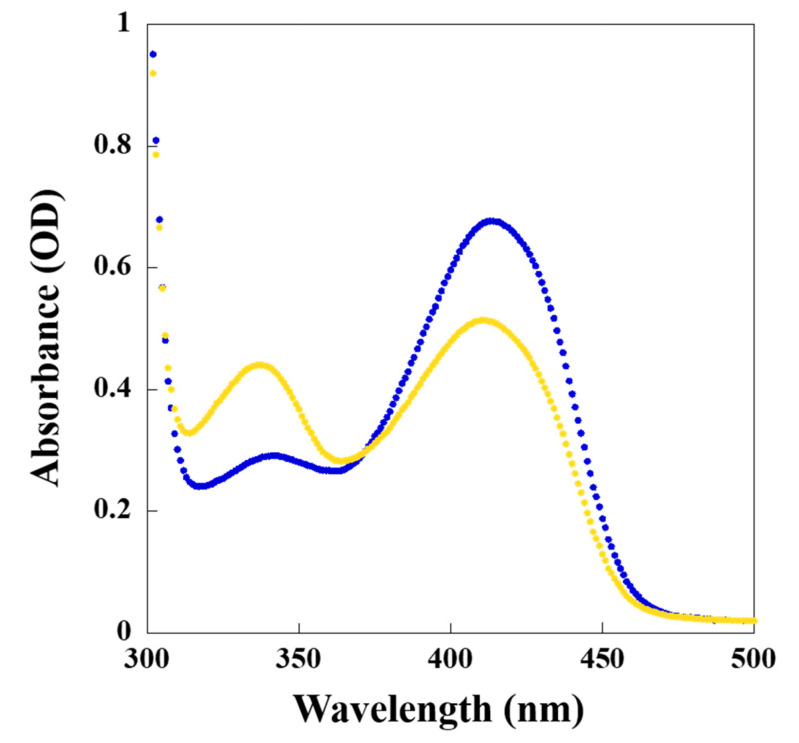
UV-visible spectrum of Slr1022 with addition of substrates. The spectrum of Slr1022 (322.9 μmol/L) alone without substrates (blue curve), 30 min after the addition of AcOrn (2 mmol/L) to Slr1022 (orange curve).

There was the highest absorption peak at a wavelength of 410 nm when the purified recombinant Slr1022 protein was scanned alone, indicating that the cofactor PLP was bound to Slr1022 in the form of internal aldimine (Figure 5) [12]. After the addition of AcOrn to recombinant Slr1022, which reacted for 30 min, the absorbance at 410 nm decreased and a new absorption peak at 340 nm appeared, which meant that PLP was converted to PMP in the first half reaction (Figure 5 and Figure S4). Due to the short reaction time (30 min), the complete conversion of PLP to PMP was not observed as that of CrmG protein reported by Xu et al. [22]. 

### 2.7. Kinetic and Catalytic Mechanism of Slr1022 as AcOAT

**Figure 6:**
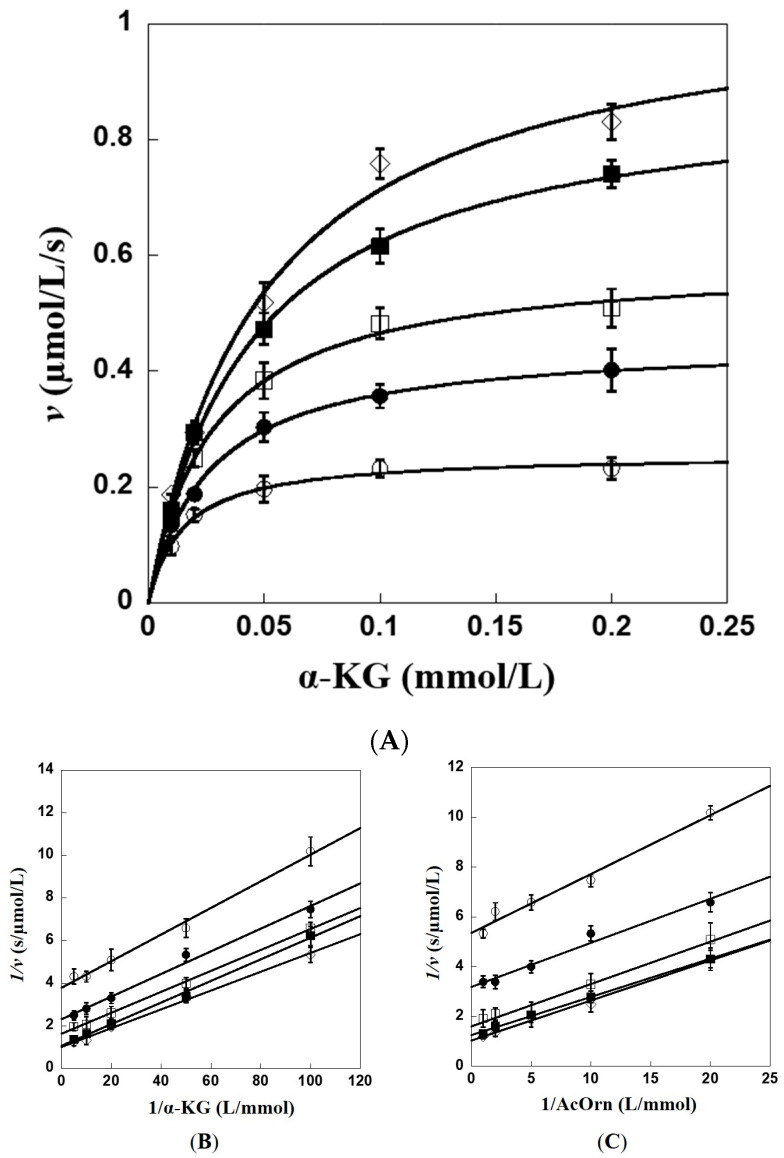
Kinetic mechanism of Slr1022 as AcOAT. (**A**): Plot of Slr1022 initial velocity vs. α-KG concentration. AcOrn concentrations: 0.05 mmol/L (○), 0.1 mmol/L (●), 0.2 mmol/L (□), 0.5 mmol/L (■), and 1 mmol/L (◇). (**B**): Double reciprocal plot of initial velocity vs. α-KG concentration. AcOrn concentrations: 0.05 mmol/L (○), 0.1 mmol/L (●), 0.2 mmol/L (□), 0.5 mmol/L (■), and 1 mmol/L (◇). (**C**): Double reciprocal plot of initial velocity vs. AcOrn concentration. α-KG concentrations: 0.01 mmol/L (○), 0.02 mmol/L (●), 0.05 mmol/L (□), 0.1 mmol/L (■), and 0.2 mmol/L (◇).

AcOAT belongs to aminotransferase family, which adopts a ping-pong catalytic mechanism [23]. At five different concentrations of substrate AcOrn, the catalytic reaction initial velocities of Slr1022 were recorded as a function of the concentrations of α-KG (Figure 6A). The data were fitted well with Michaelis–Menten equation. Double reciprocal plots were obtained from the original initial velocities vs. concentrations curves (Figure 6B,C). It was clear that the double reciprocal plots were parallel lines at the assayed concentrations for both substrates of AcOrn and α-KG, indicating that Slr1022 catalysis as AcOAT followed the ping-pong kinetic mechanism and there was no substrate inhibition for both substrates (Figure 6B,C).

**Figure 7:**
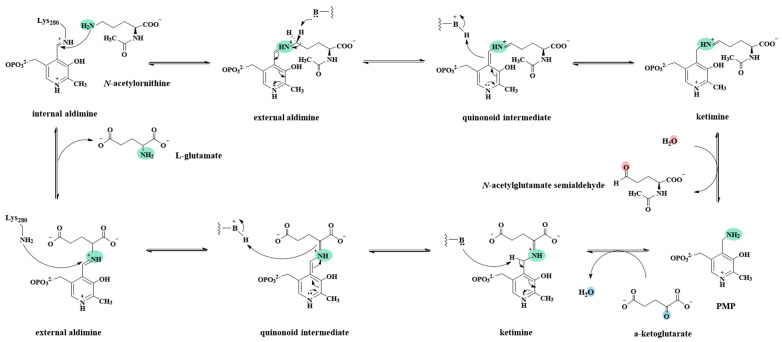
Postulated catalytic mechanism of Slr1022 as AcOAT.

The catalytic mechanisms of OAT and GABA-AT were well studied in detail [20,23,24]. AcOAT was in the same family with OAT and GABA-AT. Therefore, AcOAT catalysis proceeded in the similar way as that of OAT and GABA-AT. After combining the data of the spectral characteristics, model structure, and kinetic characterization of the Slr1022 wild-type and mutants, the catalytic mechanism of Slr1022 as AcOAT was postulated (Figure 7). Firstly, the aldehyde group of PLP was attacked by ε-amino group of Lys280 to form the enzyme-PLP Schiff base as an internal aldimine. After addition of substrate AcOrn, the δ-amino group of AcOrn nucleophilically attacked the carbon atom of the internal aldimine Schiff base to form the AcOrn-PLP Schiff base as an external aldimine and release Lys280 residue. Secondly, the δ-carbon atom of AcOrn-PLP aldimine was deprotonated to form a quinonoid intermediate, which was then transformed into a ketimine by proton transfer. Finally, the ketimine was hydrolyzed to produce *N*-acetylglutamate-5-semialdehyde and release PMP. In the second half reaction, the amino group of PMP attacked the keto carbonyl group of α-KG to form α-KG-PMP Schiff base as ketimine, which was deprotonated to form a quinonoid intermediate. This intermediate was then attacked by the ε-amino group of Lys280 in the form of an external aldimine to finish the second half reaction (Figure 7 and Figure S4).

## 3. Discussion

The ORF of *slr1022* was firstly reported to be an *argD* gene encoding an AcOAT, which played an important role in the arginine biosynthesis pathway of *Synechocystis* sp. PCC6803 [13]. Bioinformatic analysis of *Synechocystis* sp. PCC6803 genes involved in the arginine catabolism revealed that the protein encoded by *slr1022* gene might also use Orn as a substrate [14]. More recently, the *slr1022* gene of *Synechocystis* sp. PCC6803 was demonstrated to encode a GABA-AT, which closed the tricarboxylic acid cycle of *Synechocystis* sp. PCC6803 through the GABA shunt [15]. Nevertheless, the detailed kinetic characteristics of the Slr1022 protein as AcOAT, OAT, and GABA-AT were not investigated in depth. As shown in Table 1, the *K_M_* of Orn and GABA with the Slr1022 protein were 20.9 mmol/L and 78.1 mmol/L, which were 174 times and 650 times that of AcOrn with Slr1022, respectively. The catalytic efficiency of the Slr1022 protein as AcOAT was 2500-fold and 10,700-fold higher than that of Slr1022 as OAT and GABA-AT, respectively (Table 1). The difference between *K_M_* of α-KG with Slr1022 as AcOAT, OAT, and GABA-AT was not considerable (0.13 vs. 0.039 mmol/L, 3.3-fold) (Table 1). A similar situation was observed for the difference between the catalytic efficiency with regard to α-KG for Slr1022 functioning differently (8.46 × 10^2^ vs. 6.41 × 10^4^ M^−1^s^−1^, 76-fold) (Table 1). These results were quite reasonable since α-KG participated in the second half reaction of the catalysis, in which the respective substrates of AcOAT, OAT, and GABA-AT were not involved (Figure 7 and Figure S4). It is worth noting that the *k*_cat_ discrepancy between GABA and α-KG (0.14 s^−1^ vs. 0.056 s^−1^ in Table 1) was possibly caused by the sub-saturating concentration of GABA in the reaction mixture when Slr1022 functioned as GABA-AT.

The *K_M_* of AcOrn with Slr1022 was close to that of AcOrn with AcOAT from *E. coli* (0.15 mmol/L), and the catalytic efficiency (*k*_cat_/*K_M_*) of Slr1022 as AcOAT was higher than that of AcOAT from *E. coli* (4.0 × 10^3^ M^−1^s^−1^) [25]. However, the AcOAT from *Salmonella typhimurium* showed a lower *K_M_* and a higher *k*_cat_/*K_M_* of 0.037 mmol/L and 4.2 × 10^5^ M^−1^s^−1^, respectively, than those of Slr1022 [12,17]. Therefore, the Slr1022 protein mainly functioned as AcOAT with a low substrate specificity towards Orn and GABA.

The effects of metal ions on the catalytic activity of AcOAT from different sources varied significantly. In this study, Mn^2+^ enhanced the activity of Slr1022 by 38% and Ni^2+^ inhibited the activity of Slr1022 by 29%, compared to that of Slr1022 with the addition of EDTA (Figure 3A). However, Mn^2+^ strongly inhibited the activity of AcOAT from *Corynebacterium crenatum* [26] and showed a minute effect on the activity of AcOAT from *E. coli* [11]. Meanwhile, Ni^2+^ showed an activation effect on AcOATs from *Corynebacterium crenatum* and *E. coli* [11,26]. For Slr1022, Zn^2+^ showed the most inhibition effect among the tested metal ions. It remains unclear how the metal ions affect the AcOrn transaminase activity of Slr1022 yet.

The optimum reaction temperature for Slr1022 was 30 °C, which was the same case for AcOAT from *Corynebacterium crenatum* [26]. The activity of Slr1022 at temperatures of 15 °C to 55 °C varied dramatically (Figure 3B). In contrast, AcOAT from *Corynebacterium crenatum* displayed 90% of the optimal activity in the temperature range of 5–55 °C and it maintained 80% of the initial activity after keeping at 4–40 °C for 1 h [27]. These results indicated that AcOAT from *Corynebacterium crenatum* had a better thermostability than Slr1022.

Kinetic analysis revealed that both D251A and K280A mutants exhibited no catalytic activity (Table 2), indicating that these two residues were crucial for Slr1022 catalysis. Asp251 and Lys280 were highly conserved throughout the aminotransferase family [12]. It was proposed that the β-carboxyl group of the Asp residue formed hydrogen bonds or salt bridges with nitrogen atoms of the PLP pyridine ring and enhanced the function of PLP as an “electron reservoir” in the enzymatic catalysis by stabilizing the protonated nitrogen atom of the PLP pyridine ring as an external aldimine (Figure 7) [19]. Hence, the D251E mutant maintained 20% of the activity of the wild-type Slr1022, due to the carboxyl group of Glu, whereas the D251A mutant was inactive (Table 2). Similarly, the Asp222 residue of aspartate aminotransferase from *E. coli* was crucial for catalysis in that the activity of D222A and D222N was only 0.1% and 0.03%, respectively, of that of the wild-type protein, and the catalytic rate was restored to 10% of that of the wild-type for the D222E mutant [28]. The ε-amino group of the Lys residue in the active site reacted with the carbonyl group of PLP to form the Schiff base, which was the first step in the catalysis of aspartate aminotransferase and GABA-AT [29,30]. The covalent bond between Lys residue and PLP was observed in the crystal structure of AcOAT from *Mycobacterium tuberculosis* (PDB ID: 7NNC) [18]. The mutation of Lys280 to Ala280 of Slr1022 made the protein unable to form Schiff base with PLP and then could not initiate the catalytic reaction (Table 2 and Figure 7).

The mutants of E223S and E223A showed increased *K_M_* with AcOrn (65- and 73-fold higher, respectively) (Table 2), indicating that Glu223 residues functioned in the binding of AcOrn with Slr1022 protein. In the first half reaction, the salt bridge formed between Glu223 and Arg402 residues was destroyed due to the E223S or E223A mutation, and the positive charge in Arg402 could not be neutralized (Figure 4B). Therefore, Arg402 residue might compete with Arg163 residue for the α-carboxyl group of AcOrn rendering the unsuitable position of AcOrn in the active site, which consequently made the δ-amino group of AcOrn unable to react with the Schiff base formed by Lys280 and PLP (Figure 7). Consequently, the affinity of AcOrn with E223S or E223A was significantly reduced. In the second half reaction, due to the absence of β-carboxyl group in E223S or E223A residue, Arg402 was free to form a salt bridge with α-carboxyl group of α-KG, while Arg163 formed a salt bridge with γ-carboxyl group of α-KG, which correctly located α-KG to PMP. Therefore, the affinity of α-KG with the E223S or E223A variant was unaffected. Likewise, both variants of R163A and R402A made the enzyme almost unable to recognize the substrate of AcOrn resulting in extremely low catalytic efficiency (Table 2).

## 4. Materials and Methods

### 4.1. Materials

*E. coli* BL21 (DE3) strains and expression vector pET-28a were preserved in our laboratory. Site-directed mutagenesis kit and Ni-NTA resin were purchased from Beijing Transgen Biotechnology Co., Ltd. (Beijing, China). The primers and sequencing were synthesized and completed by Shanghai Xiangyin Biotechnology Co., Ltd. (Shanghai, China). Glutamate dehydrogenase (GDH) and AcOrn were purchased from Shanghai Yuanye Biotechnology Co., Ltd. (Shanghai, China), and other reagents were purchased from Beijing Solarbio Life Science Co., Ltd. (Beijing, China).

### 4.2. Expression and Purification of Recombinant Slr1022 Protein

The expression and purification of recombinant Slr1022 protein has been previously described [17]. In brief, the gene of *slr1022* encoding AcOAT from *Synechocystis* sp. PCC6803 was constructed into pET28a vector to generate the plasmid pET28a-*slr1022*, which was transformed into the *E. coli* BL21 (DE3) competent cells. Then, the transformed cells were cultured in Luria Broth (LB) liquid media with 50 μg/mL kanamycin at 37 °C and 180 rpm until the OD_600 nm_ of the media reached ca. 0.6–0.8, which was induced by isopropyl-β-D-thiogalactopyranoside (IPTG) with the final concentration of 0.2 mmol/L at 16 °C and 180 rpm for 24 h. The sonicated cell lysate was centrifuged at 4 °C and 12,000 rpm for 1 h, and thereafter the filtered supernatant containing recombinant Slr1022 flowed through Ni-NTA resin and was eluted with lysis buffer (20 mmol/L Tris-HCl, pH 7.5) containing imidazole of 20~200 mmol/L. The target protein was dialyzed over buffer containing 20 mmol/L Tris-HCl, pH 7.5, 100 mmol/L NaCl with 10% glycerol, and concentrated to desired concentration. The purity of Slr1022 was analyzed by 12% SDS-PAGE.

The site-directed mutated Slr1022 mutants were obtained with the same protocols for wild-type protein by substituting the plasmid pET28a-*slr1022* with the mutated genes, which were prepared using the commercial site-directed mutagenesis kit with pET28a-*slr1022* as template and commercial primers (Table S1).

### 4.3. Oligomer State of Recombinant Slr1022 Protein

The molecular weight of recombinant Slr1022 protein in its native state was detected by fast protein liquid chromatography (FPLC) using Bio-Rad NGC system with ENrich SEC 650 gel filtration column (the mobile phase was standard PBS buffer without exogenous PLP). Myoglobin (17 kDa), ovalbumin (44 kDa), bovine serum albumin (67 kDa), and bovine blood gamma-globulin (158 kDa) were used as the standard proteins to detect the molecular weight of the proteins by FPLC system. The relationship between molecular weight (Mw) of proteins and the retention time (RT) can be fitted with the following equation: lnMw = a − b × RT. 

### 4.4. Kinetic Characterization of Slr1022 as AcOAT

The kinetic constants of Slr1022 as AcOAT were determined by measuring the amount of NADH production with a UV spectrophotometer at 340 nm [12]. Typically, the reaction solution consisted of 6 mmol/L nicotinamide adenine dinucleotide (NAD^+^), 0.05–2 mmol/L AcOrn, 1 mmol/L α-KG, 0.1 mmol/L PLP, specific amount of recombinant Slr1022, and GDH in 100 mmol/L Tris-HCl, pH 8.5 buffer with a total volume of 500 μL at 25 °C. An appropriate amount of GDH was used to ensure that the transamination was the rate-limiting step [31]. The initial reaction velocities at different concentrations of AcOrn were calculated based on the production of NADH. The maximum reaction velocity (*V*_max_) and Michaelis constant (*K_M_*) were deduced by fitting the data into the Michaelis–Menten equation with KaleidaGraph 4.0 software. The *K_M_* of α-KG with Slr1022 was determined with the same protocols by fixing the concentration of AcOrn at 2 mmol/L and varying the concentrations of α-KG at 0.01–1.0 mmol/L. The catalytic rate (*k*_cat_) was calculated by the definition of *k*_cat_ = *V*_max_/[Slr1022]. To assay the kinetic parameters of Slr1022 variants, the concentrations of AcOrn and α-KG varied to ensure that one substrate concentration was in the range of 0.5 *K_M_*–5 *K_M_* while the other one was saturated. All experiments were repeated three times. 

### 4.5. Orn Transaminase Activity Assay of Slr1022

The catalytic function of Slr1022 as OAT was determined in the same way as that of AcOAT, except that the substrate AcOrn was replaced with Orn. In a 200 µL reaction solution, 6 mmol/L NAD^+^, 0.5 mmol/L α-KG, 2–100 mmol/L Orn, 0.1 mmol/L PLP, 9.4 μmol/L recombinant Slr1022, and appropriate GDH were added into 100 mmol/L Tris-HCl, pH 8.5 buffer at 25 °C. The absorbance values at 340 nm were continuously measured by microplate reader, and the initial reaction velocities were calculated at different Orn concentrations. Similarly, the *K_M_* of α-KG with Slr1022 as OAT was determined with Orn concentration fixed at 100 mmol/L and α-KG concentration varied at 0.02–1.0 mmol/L. The *V*_max_, *K_M_*, and *k*_cat_ of Slr1022 as OAT were deduced using the software of KaleidaGraph 4.0. All experiments were repeated three times.

### 4.6. GABA Transaminase Activity Assay of Slr1022

The catalytic function of Slr1022 as GABA-AT was determined in a similar way as reported previously with minor modifications [23]. GABA-AT catalyzes the conversion of GABA and α-KG to succinate semialdehyde and L-glutamate. Succinate semialdehyde could be catalyzed by succinate semialdehyde dehydrogenase (SSADH) to form succinate, which was accompanied by the production of NADPH. The catalytic activity of Slr1022 as GABA-AT was measured by detecting the production of NADPH at 340 nm. In a 200 µL reaction solution, 2 mmol/L Mg^2+^, 2 mmol/L nicotinamide adenine dinucleotide phosphate (NADP^+^), 0.5 mmol/L α-KG, 0.05 mmol/L PLP, 10–600 mmol/L GABA, 9.7 μmol/L SSADH from *Anabaena* sp. PCC7120 [32], and 9.4 μmol/L Slr1022 were added into 100 mmol/L Tris-HCl, pH 8.5 buffer at 25 °C. The absorbance values at 340 nm were continuously measured with a microplate reader, and the initial reaction velocities were calculated at different concentrations of GABA. The *K_M_* of α-KG with Slr1022 as GABA-AT was determined with GABA concentration fixed at 600 mmol/L and α-KG concentration varied at 0.02–1.0 mmol/L. The *V*_max_, *K_M_*, and *k*_cat_ of Slr1022 as GABA-AT were deduced using the software of KaleidaGraph 4.0. All experiments were repeated three times.

### 4.7. Effect of Metal Ions on the AcOrn Transaminase Activity of Slr1022

The effect of different metal ions on the AcOrn transaminase activity of Slr1022 was detected by discontinued method. Step 1: 500 µL reaction solution consisted of 2 mmol/L different metal ions (MgSO_4_, CaCl_2_, MnCl_2_, NiSO_4_, CoCl_2_, CuSO_4_, and ZnSO_4_), 1 mmol/L α-KG, 2 mmol/L AcOrn, 0.1 mmol/L PLP, and 0.1 µmol/L recombinant Slr1022 protein in 100 mmol/L Tris-HCl, pH 8.5 buffer at 25 °C. The reaction solution with 10 mmol/L ethylene diamine tetraacetic acid (EDTA) was used as control. The reaction was stopped after specific time by heating the reaction solution at 100 °C for 1 min, and then the reaction solution was centrifuged at 12,000 rpm for 10 min. Step 2: The 200 μL reaction mixture consisted of 40 μL supernatant from step 1, 6 mmol/L NAD^+^, and appropriate amount of GDH in 100 mmol/L Tris-HCl, pH 8.5 buffer at 25 °C. The initial and final absorbance values of the reaction mixture of step 2 at 340 nm were measured by microplate reader. The effect of different metal ions on the AcOrn transaminase activity of Slr1022 was indicated by the initial velocities of the formation of glutamate (ca. 10% conversion of α-KG to glutamate), which could be calculated according to the amount of final NADH production. The software of KaleidaGraph 4.0 was used to analyze the data. All experiments were repeated three times. 

### 4.8. Effect of Temperature on the AcOrn Transaminase Activity of Slr1022

The effect of temperature on the catalytic activity of Slr1022 as AcOAT was measured in a similar way as that of the effect of metal ions. Step 1: 500 µL reaction solution contained 1 mmol/L α-KG, 2 mmol/L AcOrn, 0.1 mmol/L PLP, and 0.1 µmol/L recombinant Slr1022 protein in 100 mmol/L Tris-HCl, pH 8.5 buffer. The reaction solution was placed at different temperatures (15 °C, 20 °C, 25 °C, 30 °C, 35 °C, 40 °C, 45 °C, 50 °C, and 55 °C) for specific time, and then heated at 100 °C for 1 min to inactivate Slr1022, followed by centrifugation at 12,000 rpm for 10 min. Step 2: same as the aforementioned protocols. The software of KaleidaGraph 4.0 was used to analyze the data. All experiments were repeated three times.

### 4.9. Structure Modeling and Model Quality Evaluation of Slr1022 

The model structure of Slr1022 was calculated by the RoseTTAFold Public Server of David Baker Lab at University of Washington in Seattle (http://robetta.bakerlab.org/, accessed on 25 October 2022) [27] and modeled by online SWISS-MODEL server with crystal structure of AcOAT from *Thermotoga maritima* (PDB ID: 2E54) as template (https://swissmodel.expasy.org/, accessed on 20 October 2022) [33]. Online protein structure analysis and verification server SAVES v6.0 (https://saves.mbi.ucla.edu, accessed on 31 October 2022) was used to evaluate the model structure quality of Slr1022. The stereochemical quality of the model structure was evaluated by Ramachandran plot generated with PROCHECK program [34] and the compatibility of the primary amino acids sequence with the 3D model structure of Slr1022 was evaluated with the program of Verify 3D [35]. The default input setting parameters were used in all of the servers and programs. The model structure was displayed by Pymol 1.3.x software. 

### 4.10. Spectroscopic Properties of Slr1022 as AcOAT 

Spectroscopic properties of Slr1022 as AcOAT were characterized as reported in the reference with difference in concentrations [12]. The absorbance of recombinant Slr1022 (322.9 μmol/L) in 100 mmol/L Tris-HCl, pH 8.5 buffer was first recorded with a microplate reader (300–500 nm). Thereafter, the substrate AcOrn (2 mmol/L) was added to the enzyme solution and reacted for 30 min to carry out wavelength scanning.

## 5. Conclusions

In this study, the kinetic constants of the Slr1022 protein from *Synechocystis* sp. PCC6803 were characterized in detail for the first time. The catalytic efficiency of Slr1022 toward AcOrn was 2500-fold and 10,700-fold higher than that toward Orn and GABA, respectively. These results proved that the Slr1022 protein mainly functioned as AcOAT with the minute activity of OAT and GABA-AT. Slr1022 displayed a moderate temperature tolerance in the range of 15–55 °C, despite showing optimal activity at 30 °C. The tested metal ions other than Zn^2+^ did not impose a considerable effect on the activity of Slr1022 as AcOAT.

AcOAT was a PLP-dependent enzyme that catalyzed the conversion of AcOrn to *N*-acetylglutamate-5-semialdehyde with the aid of α-KG as a cosubstrate. The kinetic characteristics and model structure of Slr1022 showed that Asp251, Lys280, and Gly126 residues were the key amino acids for Slr1022 catalysis. The cofactor PLP was covalently bound to the ε-amino group of Lys280 by the Schiff base to form an internal aldimine, which was the first step of the catalysis reaction. The β-carboxyl group of Asp251 might form hydrogen bonds or salt bridges with protonated nitrogen on the pyridine ring of PLP, thus stabilizing the positive charge on the protonated nitrogen and enhancing the function of PLP as an “electron reservoir” in the enzymatic catalysis process. The hydrogen bonds of the hydroxyl group of Thr308 with PLP phosphate oxygen and the amide nitrogen of Gln254 with the hydroxyl of PLP played an important role in stabilizing the PLP that was involved in the intermediates. Glu223, Tyr39, Arg163, and Arg402 residues were associated with substrate recognition and binding, and Glu223 might function as a switch between the two half reactions. Finally, these findings indicated that the catalytic mechanism of Slr1022 as AcOAT was essentially similar to the catalytic mechanisms of OAT and GABA-AT.

## Figures and Tables

**Table 1:** Apparent kinetic parameters of Slr1022 with different substrates.

Enzymatic Function (Substrate)	Respective Substrate			α-KG		
	*K_M_* (mmol/L)	*k*_cat_ (s^−1^)	*k*_cat_/*K_M_* (L/(mol·s))	*K_M_* (mmol/L)	*k*_cat_ (s^−1^)	*k*_cat_/*K_M_* (L/(mol·s))
AcOAT (AcOrn) [17]	0.12 ± 0.01	2.31 ± 0.06	1.93 × 10^4^	0.039 ± 0.004	2.50 ± 0.08	6.41 × 10^4^
OAT (Orn)	20.9 ± 3.5	0.16 ± 0.01	7.7	0.13 ± 0.03	0.11 ± 0.01	8.46 × 10^2^
GABA-AT (GABA)	78.1 ± 18.7	0.14 ± 0.01	1.8	0.04 ± 0.01	0.056 ± 0.003	1.4 × 10^3^

**Table 2:** Apparent kinetic parameters of Slr1022 wild-type and mutants.

Proteins	*K_M_*_AcOrn_ (mmol/L)	*K_M_*_α-KG_ (mmol/L)	*k*_cat_ (s^−1^)	Relative Catalytic Rate (% of Wild-Type)
wild-type [17]	0.12 ± 0.01	0.039 ± 0.004	2.31 ± 0.06	100
T308A	0.14 ± 0.02	0.025 ± 0.003	0.007 ± 0.002	0.3
Q254A	0.11 ± 0.01	0.04 ± 0.01	0.02 ± 0.0005	0.9
S125A	0.23 ± 0.02	0.065 ± 0.008	0.32 ± 0.01	14
A127S	0.59 ± 0.12	0.09 ± 0.02	0.21 ± 0.01	9.1
D251E	1.39 ± 0.22	0.039 ± 0.006	0.47 ± 0.03	20
D251A	NA	NA	NA	NA
K280A	NA	NA	NA	NA
G126A	NA	NA	NA	NA
E223S	8.81 ± 2.96	0.015 ± 0.004	3.67 ± 0.43	160
E223A	7.77 ± 1.76	0.15 ± 0.02	1.17 ± 0.09	50
R163A	493 ± 108	0.92 ± 0.19	0.22 ± 0.02	9.5
R402A	250 ± 32	0.87 ± 0.14	0.41 ± 0.02	18
Y39F	0.47 ± 0.05	0.08 ± 0.03	0.17 ± 0.01	7.4

NA: No activity.

## Data Availability

The data presented in this study are available in the manuscript and in the Supplementary Materials.

## Supplementary Materials

The following supporting information can be downloaded at: https://www.mdpi.com/article/10.3390/ijms24065853/s1.
