# Supplementary material for: Kinetic Characterization and Catalytic Mechanism of N-Acetylornithine Aminotransferase Encoded by slr1022 Gene from Synechocystis sp. PCC6803
Source: Int J Mol Sci. 2023 Mar 19;24(6):5853. doi: 10.3390/ijms24065853 (PMC10057298; doi:10.3390/ijms24065853)
Supplement: Supplementary file 1 [file ijms-24-05853-s001.zip › ijms-2252758-supplementary.pdf]

Kinetic Characterization and Catalytic Mechanism of *N*-Acetylornithine Aminotransferase Encoded by *slr1022* Gene from *Synechocystis* sp. PCC6803

Table S1. Primer sequences of Slr1022 mutants

Mutants	Primers	Sequences of Primers
A127S	A127S-F	CTTTTGCAACTCTGGG**T**CAGAGGCCAAT
	A127S-R	**A**CCCAGAGTTGCAAAAGAAAACCCGGTC
D251A	D251A-F	ATATTTTGTTGGTCTTT**G**CCGAAGTGCAAG
	D251A-R	**G**CAAAGACCAACAAAATATCGTTTTGGTCAC
G126A	G126A-F	TTTTCTTTTGCAACTCTG**C**GGCAGAGGCCAAT
	G126A-R	**G**CAGAGTTGCAAAAGAAAACCCGGTCCGCACA
K280A	K280A-F	ACATTTTCACCAGTGCC**G**CGGGTCTGGCCG
	K280A-R	**G**CGGCACTGGTGAAAATGTCTGGCTCCACC
Q254A	Q254A-F	GGTCTTTGACGAAGTG**GCG**GTGGGGGTAG
	Q254A-R	**CGC**CACTTCGTCAAAGACCAACAAAATATCGT
S125A	S125A-F	GTTTTCTTTTGCAAC**G**CTGGGGCAGA
	S125A-R	**C**GTTGCAAAAGAAAACCCGGTCCGCA
T308A	T308A-F	GGCAACCATGCCAGT**G**CCTTTGGTGGTAAT
	T308A-R	**C**ACTGGCATGGTTGCCCGGCTCAAATA
E223A	E223A-F	AACCTCTCCAAGGGG**C**GGGGGGAGTC
	E223A-R	**G**CCCCTTGGAGAGGTTCGAGGAAAATGGCTG
E223S	E223S-F	GAACCTCTCCAAGGG**AGC**GGGGGAGTCC
	E223S-R	**GCT**CCCTTGGAGAGGTTCGAGGAAAATGGCTG
D251E	D251E-F	TTTTGTTGGTCTTTGA**A**GAAGTGCAAG
	D251E-R	**T**TCAAAGACCAACAAAATATCGTTTTGGTC
Y39F	Y39F-F	CCTATGTGATGAACACCT**T**TGGGCGATTTC
	Y39F-R	A**A**AGGTGTTCATCACATAGGTATCAAAATCTG
R163A	R163A-F	TAGTTTCCACGGC**GCG**ACCCTAGCCAC
	R163A-R	**CGC**GCCGTGGAAACTAGCTTTGGCG
R402A	R402A-F	TGGTCCCAAAGTGTTA**GCG**TTTGTGCCCC
	R402A-R	**CGC**TAACACTTTGGGACCAGCGGGGG

Note: The mutated nucleotides are in bold and underlined.

Table S2. Ramachandran plot statistics of Slr022 model structure computed with PROCHECK program

Residue regions	Proportion (%)
residues in most favored regions	86.2
residues in additional allowed regions	12.9
residues in generously allowed regions	0.3
residues in disallowed regions	0.6
non-glycine and non-proline residues	100.0

A

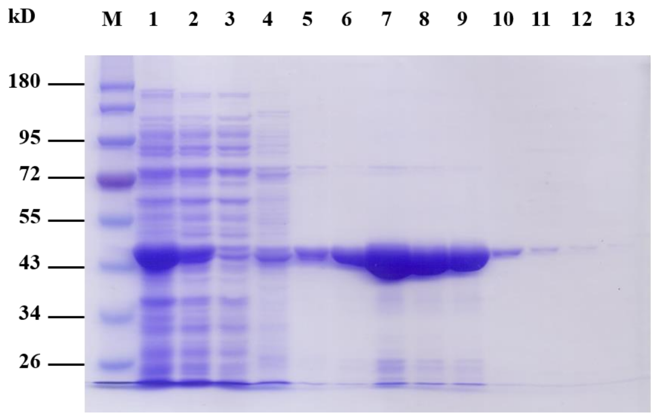

C

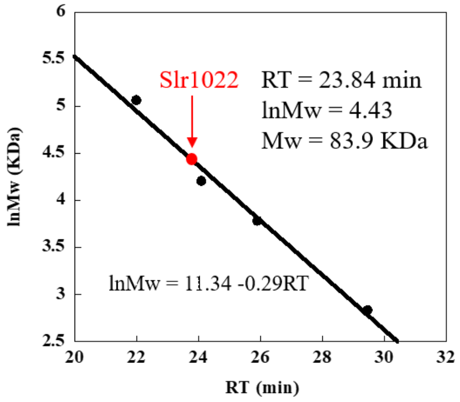

B

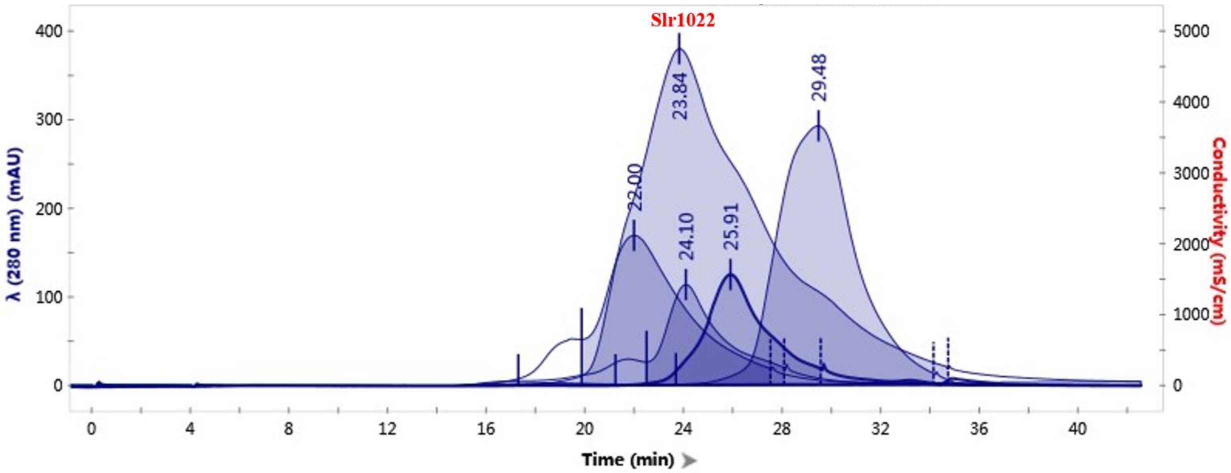

Figure S1. SDS-PAGE and fast protein liquid chromatography of Slr1022 protein. **A**: SDS-PAGE of purification of Y39F mutant. **B**: The elution peaks are bovine blood γ-globulin (158 kDa), Slr1022 protein, bovine albumin (67 kDa), albumin egg (44 kDa), myoglobin (17 kDa) from left to right in order and the value on the peak indicates the retention time (RT) of the protein in the high-resolution gel column. **C**: The standard curve was fitted by the formula $\ln M_w$ = 11.34-0.29RT. Therefore, the size of Slr1022 protein was calculated to be about 83.9 kDa.

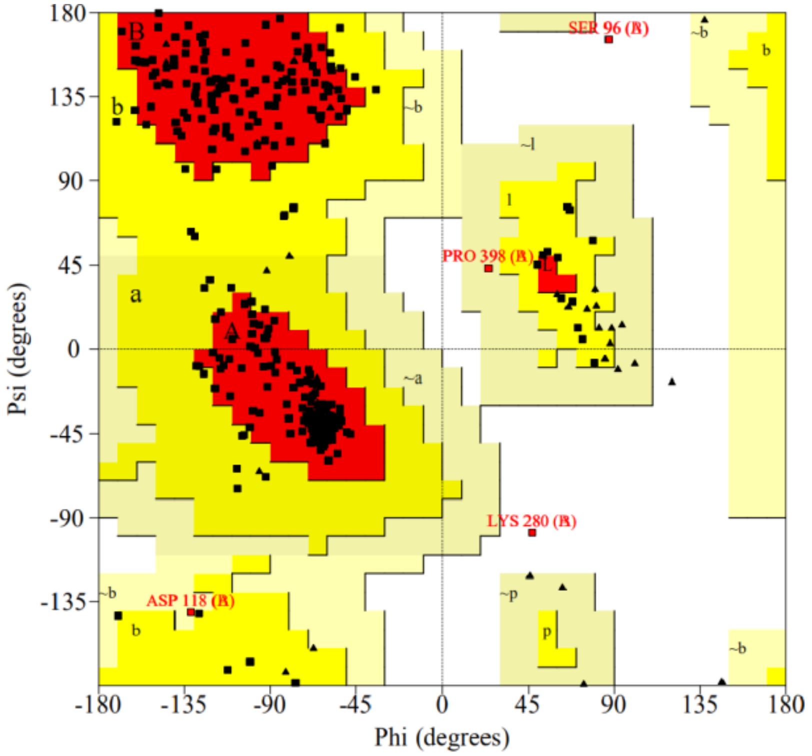

Figure S2. Ramachandran plot of Slr1022 model structure generated by PROCHECK program.

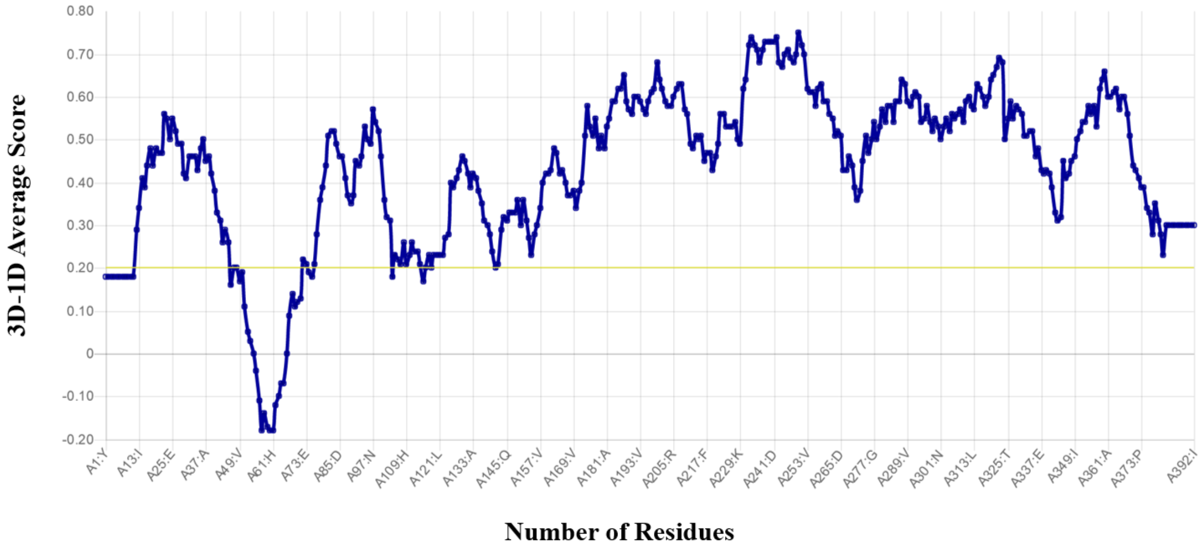

Figure S3. Verify 3D score chart of Slr1022. 89.92% of the amino acids have scored >= 0.2.

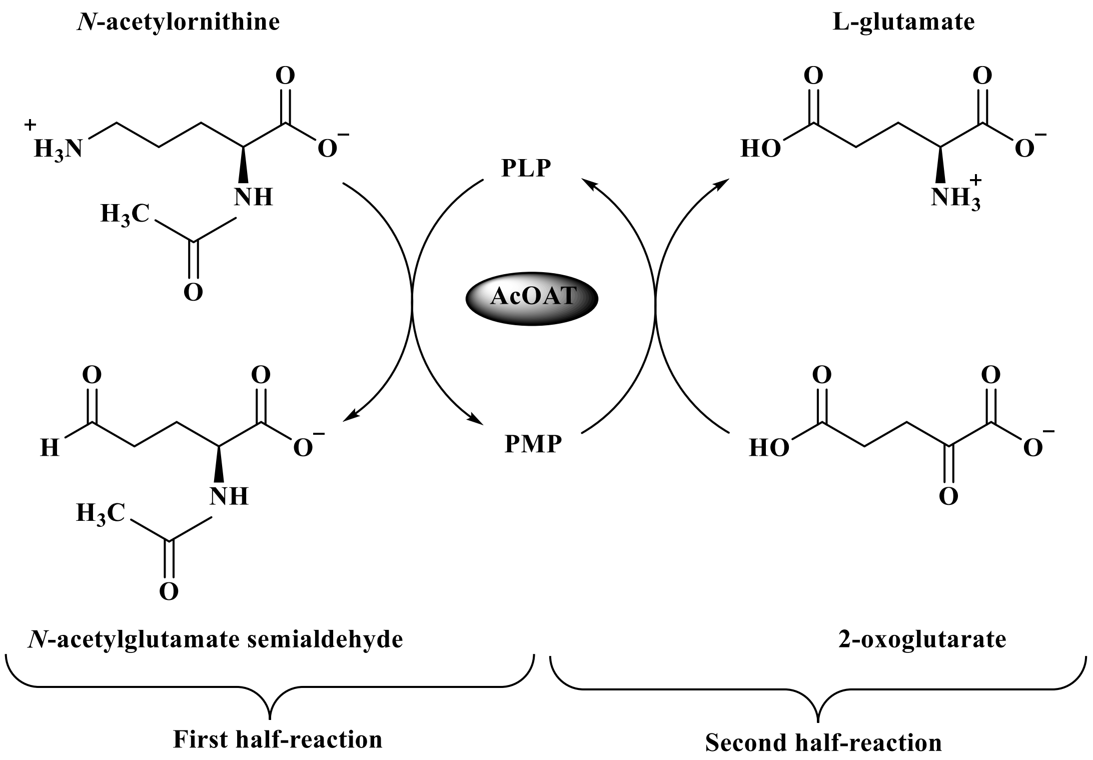

Figure S4. Both half reactions of Slr1022 as AcOAT. AcOAT, *N*-acetylornithine aminotransferase; PLP, pyridoxal 5′-phosphate; PMP, pyridoxamine 5′-phosphate. This scheme was adapted from reference [1,2].

[1] H. Lee, J.I. Juncosa, R.B. Silverman, Ornithine aminotransferase versus GABA aminotransferase: implications for the design of new anticancer drugs, Medicinal Research Reviews 35 (2015) 286-305. 10.1002/med.21328.

[2] V. Rajaram, P. Ratna Prasuna, H.S. Savithri, M.R. Murthy, Structure of biosynthetic N-acetylornithine aminotransferase from Salmonella typhimurium: studies on substrate specificity and inhibitor binding, Proteins 70 (2008) 429-441. 10.1002/prot.21567.
